# Impaired Sleep, Circadian Rhythms and Neurogenesis in Diet-Induced Premature Aging

**DOI:** 10.3390/ijms18112243

**Published:** 2017-10-26

**Authors:** Alexander J. Stankiewicz, Erin M. McGowan, Lili Yu, Irina V. Zhdanova

**Affiliations:** 1 Department of Preclinical Research and Development, BioChron LLC, Worcester, MA 01605, USA; alexander.stank@gmail.com (A.J.S.); lilifish1964@gmail.com (L.Y.); 2 Department of Anatomy and Neurobiology, Boston University School of Medicine, Boston, MA 02118, USA; erinmcgowan20@gmail.com

**Keywords:** premature aging, circadian, neurogenesis, sleep, high caloric intake, cell cycle, scoliosis, anxiety, diurnal vertebrate

## Abstract

Chronic high caloric intake (HCI) is a risk factor for multiple major human disorders, from diabetes to neurodegeneration. Mounting evidence suggests a significant contribution of circadian misalignment and sleep alterations to this phenomenon. An inverse temporal relationship between sleep, activity, food intake, and clock mechanisms in nocturnal and diurnal animals suggests that a search for effective therapeutic approaches can benefit from the use of diurnal animal models. Here, we show that, similar to normal aging, HCI leads to the reduction in daily amplitude of expression for core clock genes, a decline in sleep duration, an increase in scoliosis, and anxiety-like behavior. A remarkable decline in adult neurogenesis in 1-year old HCI animals, amounting to only 21% of that in age-matched Control, exceeds age-dependent decline observed in normal 3-year old zebrafish. This is associated with misalignment or reduced amplitude of daily patterns for principal cell cycle regulators, *cyclins A* and *B*, and *p20*, in brain tissue. Together, these data establish HCI in zebrafish as a model for metabolically induced premature aging of sleep, circadian functions, and adult neurogenesis, allowing for a high throughput approach to mechanistic studies and drug trials in a diurnal vertebrate.

## 1. Introduction

High caloric intake (HCI) can result from an overall increase in the amount of food consumed or excess of high-calorie ingredients in dietary products, sugars, or fats. Epidemiological and experimental studies provide strong evidence of the negative impact of HCI on principal body functions. Accumulation of excessive body fat with its powerful energy-generating and endocrine effects, altered liver metabolism, cholesterol imbalance, and other effects of HCI are implicated in the development of metabolic syndrome, type 2 diabetes, and cardiovascular disorders [1,2,3,4]. These metabolic changes also constitute a high risk for sleep disorders, including insomnia and sleep apnea, and is associated with circadian rhythm abnormalities [5,6]. The most powerful evidence of the negative physiological role of HCI comes from aging studies. Caloric restriction is found to slow down aging, increase lifespan, and counteract age-related diseases [7,8,9,10].

An increasingly recognized role of sleep and circadian disorders in metabolic dysfunctions is supported by a significant association between reduced sleep duration and metabolic or cardiovascular disorders [11,12,13,14,15,16,17], and positive correlation between insomnia and altered glucose metabolism [18,19]. Furthermore, the association of mutations in core circadian clock genes with metabolic phenotypes, hyperglycemia, hyperinsulinemia, obesity, and type 2 diabetes has been reported in both animals and humans [20,21,22,23,24,25,26]. The data collected in both day-active drosophila and night-active mice suggest that the positive effects of caloric restriction on longevity can be mediated through the circadian clock-dependent mechanisms [27,28]. This is especially interesting in view of the inverse temporal relationship between core circadian mechanisms and sleep, or metabolic functions in such species [29,30]. While daily patterns of expression for core clock genes, nighttime melatonin secretion, or peak neuronal activity in the master clock are largely conserved in diurnal and nocturnal vertebrates, their sleep and metabolic processes peak at opposite phases of the daily cycle. For this reason, studying the mechanisms that are common to circadian, sleep and metabolic processes can benefit from diurnal animal models.

A rapidly developing research area of major societal importance is a link between metabolic and neurodegenerative disorders, when considering their epidemic-like rise in aging populations of industrialized countries. Both epidemiological and animal studies implicate HCI as a prominent risk factor for the development of the Alzheimer’s and Parkinson’s diseases [31,32,33,34]. While interaction between these two types of human conditions can occur due to a variety of factors, alterations in adult neurogenesis is one of the candidate links. A lack of new neurons or their inability to incorporate into existing neural circuits can be one of the significant contributions to neurodegenerative process or a failure in compensatory mechanisms allowing its progression. The role of HCI and specific high caloric diets in modulating adult neurogenesis thus attracts substantial attention. In nocturnal rodents, caloric restriction was found to increase cell proliferation, cell survival and neuronal fate in adult hippocampus, and enhance spatial learning and cognitive performance [35,36,37,38,39]. In contrast, a high intake of dietary fat can negatively affect hippocampal neurogenesis even in the absence of increased body weight [40]. Food supplements and specific dietary products, such as caffeine or ethanol, can also negatively or positively affect adult neurogenesis in species studied, and this requires further in-depth investigation [41].

Zebrafish is a popular model in developmental biology, genetics and, more recently, in neurobiology. It is a diurnal vertebrate with a robust circadian clock system and conserved sleep mechanisms [42,43]. Similar to humans, zebrafish exhibit gradual senescence [44]. Their aging phenotype includes anatomical features, such as scoliosis and muscular atrophy [45], decrease in regenerative capacity of peripheral tissues [46], development of cataracts, and an increase in senescence-associated β-galactosidase [47]. We find that aging zebrafish show a progressive reduction in locomotor activity, an increase in anxiety-like behaviors [48], a decline in circadian rhythmicity, and spatial memory [49].

Here, we show that, in zebrafish, chronic caloric excess from early age leads to premature aging reflected in multiple abnormalities, from anatomical deformities to early onset of anxiety-like behavior, altered sleep, reduced amplitude of circadian rhythms, and a remarkable decline in adult neurogenesis. Together, this establishes zebrafish as a model for metabolically induced premature aging of integrating systems and brain functions. Gradual senescence and well-appreciated high throughput capabilities of this diurnal vertebrate model provide novel opportunities for translational research and drug development.

## 2. Results

### 2.1. High Caloric Intake Leads to Increase in Brain Volume, Body Weight, Scoliosis, Anxiety-Like Behavior and High Mortality Rate

**Figure 1:**
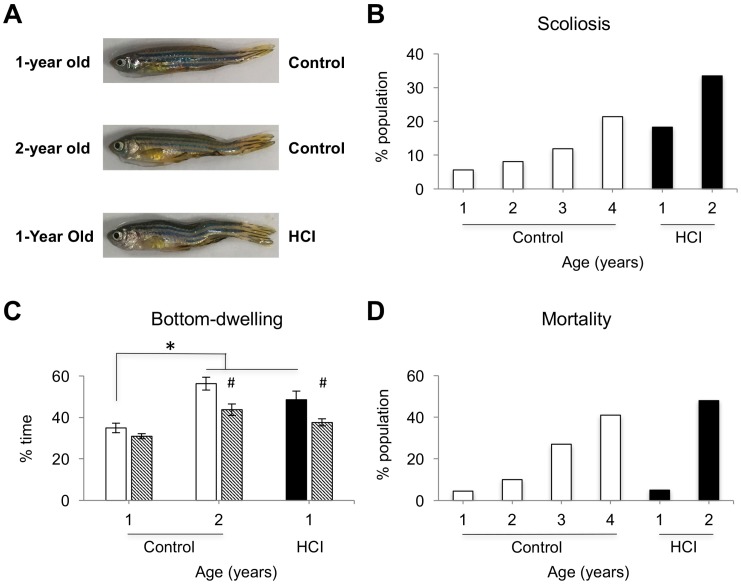
Premature aging phenotype in high caloric intake (HCI) zebrafish: anatomical and behavioral changes, and increased mortality. (**A**) Images of 1-year old Control, 2-year old Control and 1-year old HCI fish with scoliosis. (**B**) Percent of fish with scoliosis within each condition; white bar—Control fish, 1–4 years of age, black bar—HCI fish, 1–2 years of age. (**C**) Bottom-dwelling, time spent swimming at the bottom third of the tank, following no treatment (solid bar) or after Diazepam administration (diagonal stripes), *n* = 6–8 fish per group, mean ± SEM, * *p* < 0.05 vs. 1-year old Control; ^#^ *p* < 0.05 Diazepam vs. no treatment. (**D**) Mortality rate in 1–4 year old Control and 1–2 year old HCI fish (see Section 4.1. for the size of each population).

Consistent with earlier reported morphological changes in aging zebrafish [44,45], a gradual increase in scoliosis was documented in normally aging wild-type zebrafish. The rate of scoliosis, being 5.6% in 1-year old Control animals, gradually increased with aging, reaching 8.1% by two years of age, 11.9% by three years of age, and 21.4% by four years of age. Unlike that, zebrafish raised on high caloric intake (HCI) diet displayed early onset of scoliosis, observed in 18.3% of animals by the time they reached one year of age and 33.5% in 2-year old HCI fish (Figure 1A,B). See each population size in Section 4.1.

Zebrafish display a lifelong growth, as typical of other teleosts, though at the end of an individual’s lifespan muscle wasting often contributes to a decline in body mass [45,50]. The estimated brain volume (see Section 4.6. was significantly higher in 1-year old HCI fish, as compared to age-matched Control (1.62 mm^3^ ± 0.05 vs. 1.17 mm^3^ ± 0.05; *p* < 0.05, *n* = 10 for each group)). In Control animals, the body weight increased 12.1 ± 2.82% from 1–2 years of age (*p* < 0.05). HCI lead to significant increase in body weight by 1-year of age (27.9 ± 3.29%, *p* < 0.05 vs. age-matched Control), but no significant change between 1- and 2-year old HCI fish was documented.

An increase in the amount of food provided to HCI animals led to changes in feeding behaviors. The diet used in this study (Gemma-300) initially floated on the water surface, where it was readily consumed by both Control and HCI fish of different age, and was not affected by scoliosis. Within the first 20 min, food particles sank to the bottom of the tank and some fish continued feeding. On average, HCI fish spent more time feeding after each food presentation (27 ± 5.31% above age-matched 1-year old Control; *p* < 0.05). Visual inspection of food leftovers on the bottom of the tanks suggested that most of the food available was consumed by both Control and HCI fish within an hour of its presentation.

Locomotor activity in zebrafish declines with aging, and this can be reflected in both mean activity levels and changes in high-speed locomotion [48,51]. HCI led to an early onset of reduction in high-speed activity during light period (*p* < 0.05), though no significant change in total daytime activity was documented in 1-year old HCI fish. Tendency to swim close to the bottom of the tank and tank walls, i.e., bottom-dwelling and thigmotaxis, are known to be associated with anxiety-like states in zebrafish [52,53,54,55]. Recently, we reported gradual increase in such behaviors in zebrafish throughout aging and its significant increase in young animals with a knockout of dopamine transporter [48]. Similarly, the 1-year old HCI fish had a significant increase in bottom-dwelling, compared to age-matched Control, reaching levels similar to those in the 2-year old Control animals (Figure 1C). It should be noted that all of these animals, including young and aged HCI fish, can easily rise to the top of the tank and swim there over prolonged periods of time during feeding, acquiring floating food pellets. Similar to that demonstrated earlier in zebrafish experiencing cocaine-induced anxiety [53], bottom-dwelling was significantly reduced in HCI and aged animals following administration of a non-sedative dose of diazepam (Figure 1C).

The mortality was also dramatically affected by HCI. Although low mortality was documented in 1 year old HCI or Control fish, by 2-years of age the mortality rate was dramatically higher in HCI animals than in age-matched Control and similar to that for the 4-year old Control population (Figure 1D).

### 2.2. Chronic High Caloric Intake Alters Circadian Rhythms and Sleep

**Figure 2:**
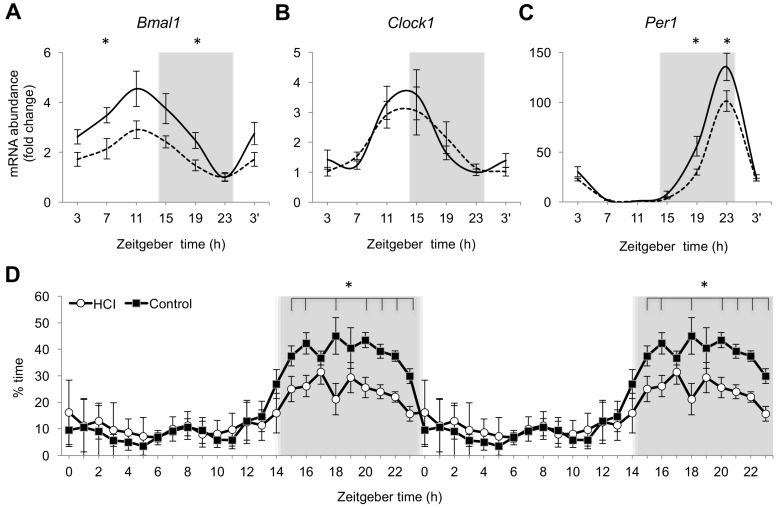
Reduction in nighttime sleep and expression of core clock genes in diet-induced premature aging. (**A**–**C**) Fold change in mRNA abundance over a 24-h period for the core clock genes (*Bmal1*, *Clock1*, *Per1*) in 1-year old HCI and Control fish, relative to within-group minimal expression. *n* = 5–6 fish per time point, per group. Solid line—Control, dashed line—HCI. (**D**) Double-plot of percent time spent in sleep over a 24-h period. Black circle—Control, open circle—HCI. *n* = 8 fish per group. In (**A**–**D**): Mean ± SEM, * *p* < 0.05 1-year old HCI vs. 1-year old Control at specific time points. Grey background—night, 14:10 Light-dark (LD) cycle. Zeitgeber time (ZT) of sample collection, ZT0 = lights-on time.

To explore an impact of chronic HCI on the circadian system, we studied the expression of core clock genes in brain tissue at 4-h intervals throughout a 24-h period, scheduled two months after these fish were maintained on a regular diet. When compared to age-matched Control, HCI animals demonstrated similar daily patterns of entrainment of gene expression to the 14:10 light-dark cycle (Figure 2A–C). The acrophase remained similar between the two groups for all three of the parameters. The peak of mRNA abundance in HCI fish corresponded to normal zeitgeber times, ZT11 for *Bmal1*, between ZT11 and ZT15 for *Clock1* and ZT23 for *Per1*. However, in HCI fish, the daily amplitude of gene expression for *Bmal1* and *Per1* was reduced (*p* < 0.05) when compared to age-matched Control. This was also reflected through significant changes in the area under the curve (AUC) for *Bmal1* and *Per1* (*p* < 0.05 for either, vs. Control), as well as at individual time points for these two genes, but not for *Clock1* (Figure 2A–C).

HCI fish demonstrated typical of diurnally-active zebrafish increase in nighttime sleep (Figure 2D). However, when compared to age-matched Control, the percent of time HCI fish spent asleep at night was significantly reduced (*p* < 0.05). The difference remained significant over the majority of the nighttime hours (Figure 2D). No significant change in the percent of daytime sleep was observed in these animals, resulting in total sleep duration over a 24-h period being significantly lower in HCI than in Control fish (*p* < 0.05). The amplitude of the daily sleep rhythm was significantly reduced in HCI (*p* < 0.05), while the acrophase remained similar to Control (Figure 2D).

### 2.3. Adult Neurogenesis Is Attenuated in Premature Aging Induced by High Caloric Intake

**Figure 3:**
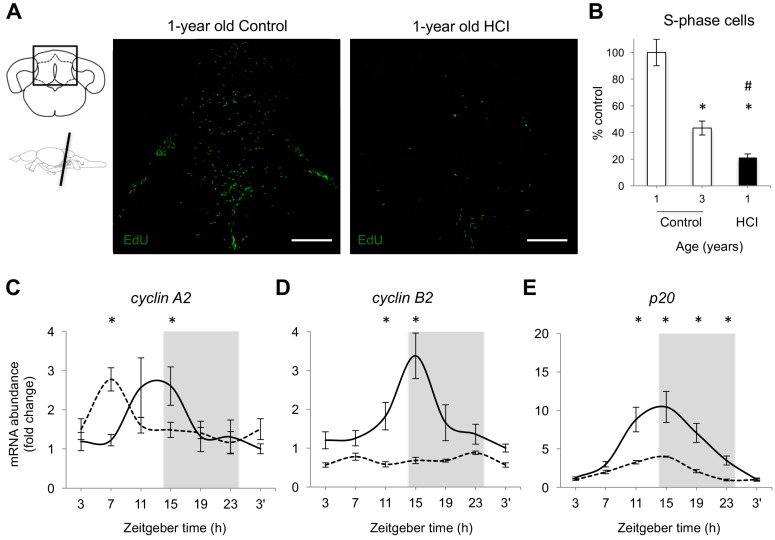
Chronic high caloric intake inhibits adult neurogenesis and expression of cell cycle regulators in brain tissue of 1-year old zebrafish. (**A**) **Left**: schematics showing cerebellar sections with boxes identifying areas presented in the images along with schematic of zebrafish brain identifying coronal planes at which the brain was cut; **Right**: representative images of the total number of 5-Ethynyl-2′-deoxyuridine (EdU) positive cells (S-phase), green, in Control and HCI fish at ZT11, following 2-h EdU exposure, intraperitoneal (i.p.) injection, Scale: 180 μm. (**B**) Percent cells in S-phase in 3-year old Control (white bar) and 1-year old HCI (black bar), relative to mean levels in 1-year old Control (100%, white bar); * *p* < 0.05 vs. 1-year old Control and ^#^ *p* < 0.05 vs. 3-year old Control; (**C**–**E**) Daily patterns of mRNA abundance for *cyclins A2* and *B2*, and *p20* in 1-year old Control (solid line) and HCI fish (dashed line); *n* = 5–6 fish per each data point; mean ± SEM, * *p* < 0.05 HCI vs. Control at specific time points. Grey background—night, 14:10 LD cycle. Zeitgeber time of sample collection, ZT0 = lights-on time.

To evaluate adult neurogenesis in HCI animals and to compare its levels to those in Control, we exposed fish to a thymidine analog, 5-Ethynyl-2′-deoxyuridine (EdU), and collected brain tissue at intervals thereafter to evaluate the number of cells undergoing S-phase of the cell division cycle (CDC). The results are illustrated in Figure 3A, showing EdU-stained cells in the largest neurogenic niche in zebrafish, the cerebellar niche. The total number of EdU-positive cells in the brain was significantly reduced in HCI animals when compared to age-matched Control (Figure 3B). Remarkably, the extremely low adult neurogenesis in HCI fish, amounting to about 21% of that in age-matched Control, was also significantly lower than in aged 3-year old Control animals (Figure 3B).

We have recently established the presence of circadian patterns of cell proliferation in individual neurogenic niches of zebrafish brain [56]. Those were reflected in both the variation in the number of S-phase cells at different times of day and the expression patterns for the principal regulators of the CDC. Here, we compared the daily patterns of expression for three such regulators between HCI fish and aged matched 1-year old Control (Figure 3C–E). In HCI animals, the *cyclin A2* expression pattern was significantly advanced, with the phase shift in acrophase being estimated based on the sine wave fit reaching 5.1 h (Figure 3C). In contrast, the *cyclin A2* daily amplitude remained similar to Control. The circadian pattern for *cyclin B2* expression was abolished in HCI fish (Figure 3D). The amplitude of expression for *p20* was significantly reduced in HCI animals (*p* < 0.0001), while the acrophase remained similar to Control (Figure 3E).

### 2.4. Age-Dependent Changes in Nighttime Sleep and Gene Expression in Zebrafish Brain

**Figure 4:**
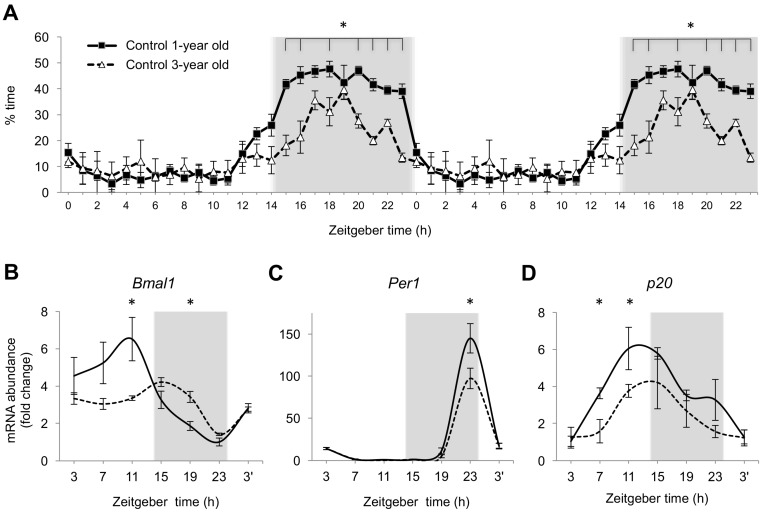
Age-dependent changes in nighttime sleep and the expression of core clock genes and cell cycle regulator in 3-year old zebrafish undergoing normal aging. (**A**) Double-plot of percent time spent in sleep over a 24-h period. Black circle—1-year old Control, open triangle—3-year old Control. *n* = 12 fish per group. (**B**–**D**) Fold change in mRNA abundance over a 24-h period for the core clock genes (*Bmal1*, *Per1*) and CDC regulator, *p20*, in 1-year old (solid line) and 3-year old (dashed line) Control fish, relative to within-group minimal expression, *n* = 5–6 fish per time point per group. In (**A**–**D**): Mean ± SEM, * *p* < 0.05 3-year old vs.1-year old Control at each time point. Grey background—night, 14:10 LD cycle. Zeitgeber time of sample collection, ZT0 = lights-on time.

To determine whether normal aging is also associated with changes in entrained circadian rhythms of sleep and the expression of clock genes or CDC regulators, we conducted similar observations in 3-year old zebrafish. Indeed, the nighttime sleep was diminished in aged animals, with no change in daytime sleep levels (Figure 4A). Similar to our earlier observations of the reduced amplitude of expression for clock genes in the eye tissue of aged zebrafish [51], reduction in *Bmal1* and *Per1* expression was now documented in the brain of 3-year old animals (Figure 4B,C). No significant change was observed in the expression of *Clock1*, *cyclins A2*, and *B2*. The expression of *p20* was lower in aged zebrafish (Figure 4D). Together, a number of changes observed in normally aging fish were similar to those in HCI animals, further supporting a hypothesis that HCI leads to premature aging.

## 3. Discussion

Over the past 100 years, the remarkable progress in biomedical research and clinical practice has significantly extended average human lifespan. However, the relatively recent obesity epidemic is a powerful counterforce to this positive dynamic [57]. Obesity heightens the morbidity and mortality risk at any age [58,59]. An increase in the overall food intake and change in food content, along with sedentary lifestyle, are considered to be the main extrinsic driving forces of this epidemic, which has now spread to children [60,61]. The extent to which the early onset of high caloric intake (HCI) interferes with maturation and aging, and the age at which normal diet, exercise, or other therapeutic interventions could reverse negative effects of HCI, remain to be fully addressed. The use of high throughput vertebrate models can help in elucidating these issues.

Here we report that in a diurnal vertebrate, zebrafish, chronic high caloric intake from the early age to maturity interferes with multiple body functions, and leads to early mortality. We find that HCI is associated with early onset of alterations in the skeletomuscular system, increased anxiety-like behavior, reduced nighttime sleep, and low amplitude of expression for core clock genes. The most remarkable HCI-induced changes are observed in adult neurogenesis, reflected in the extremely low number of replicating cells in neurogenic niches and a decline in the expression of cell cycle regulators in the brain. Importantly, these changes manifest even after a 2-month long exposure to normal diet, indicating their chronic nature. When considering that HCI phenotype in chronologically young animals includes a complex of anatomical, physiological, and behavioral changes typical of aged zebrafish, as documented here and in earlier reports [46,48,49,50,51], we suggest that HCI fish can be considered a new animal model of diet-induced premature aging.

Zebrafish is an outstanding vertebrate model for studying gradual aging, the type of aging that is characteristic of humans [44]. They have rapid development, becoming an active hunter by six days post-fertilization and reaching sexual maturity by 3 months of age. Under optimal conditions, zebrafish reach peak shape by one year of age, with close to maximal size, high fecundity, robust sleep-wake cycle, and high cognitive performance [48,49,50,51]. By two years of age, however, many zebrafish start displaying physiological and behavioral signs of aging. We find the mortality rate in zebrafish to dramatically increase by three years of age and beyond, when compared to 1-year old adults. Nevertheless, under favorable laboratory conditions, many zebrafish live for five years and, less frequently, for up to seven years. As a result, this vertebrate allows us to study the gradual progression of at least a 4-year long aging process, while monitoring the contribution of different intrinsic and extrinsic factors to “unsuccessful” or “successful” aging.

Our finding that overfeeding with regular zebrafish food leads to acceleration of the aging process adds to a large body of literature on the link between metabolism and aging [62]. Complex reciprocal interactions between metabolic processes and other physiological systems that show an age-dependent decline in function raise interesting questions on the cause-effect relationship between them, and their individual or joint contribution to premature aging. One example is the growing number of reports suggesting that alterations in the circadian clock system can both accelerate aging and cause metabolic abnormalities [63,64,65]. This is consistent with the circadian clock, defining mutual alignment and thus the effective coordination of critical body functions with each other and with periodically changing environments. Specific to metabolism, the clock is in control of the temporal patterns of behavioral rhythms of food intake, activity of digesting organs, sensitivity of cells to glucose, and efficiency of other metabolic pathways [66]. Our finding of reduced daily amplitude of expression for core clock genes in HCI fish suggest that chronic metabolic load can, in turn, lead to clock malfunction. Notably, in spite of the lower daily amplitude of expression in brain tissue, the clock genes maintain their normal temporal alignment with the light-dark cycle in HCI zebrafish. This is likely to reflect a high sensitivity of the circadian clock in zebrafish to the principal environmental time cue, ambient light [67].

Further studies are needed to provide a detailed metabolic portrait of the HCI animals, contribution of specific nutrients present in Gemma or Artemia food sources that might play significant role in HCI effects, and to determine at which age the pathological changes are initiated, including those affecting the circadian system. Our preliminary data indicate that mortality rate remains high in 2-year old HCI fish even if they are transferred to normal diet after reaching one year of age, suggesting that irreversible pathological changes might occur before that age. Interestingly, it has been reported earlier that time-restricted feeding (TRF) can be protective, when compared to the negative effects of ad libitum feeding throughout the day [68]. However, in the present study, the described pathological HCI conditions developed in spite of twice-a-day TRF, with the food being consumed by the Control and HCI fish within an hour after administration.

Sleep, one of the most robust circadian body rhythms and a critical homeostatic mechanism assuring functional integrity, also emerges as a significant modulator of morbidity and contributor to the aging process [69]. The sleep process in zebrafish was first characterized in 2001, establishing its regulation by the principal hormone of the circadian system, melatonin, and comparing melatonin effects to those of the commonly used hypnotic medications [70]. Further research in this area highlighted a conserved nature of sleep regulation in zebrafish [43,71], including the role of such sleep modulators as orexin/hypocretin and histamine that play major roles in humans’ sleep-activity cycle [72,73,74,75,76,77]. Reduction in the duration of nighttime sleep in HCI fish is analogous to the decline in sleep duration and efficiency typically observed in aging humans [69]. Interestingly, we do not find a compensatory increase in daytime sleep duration in HCI zebrafish, leading to their total sleep time being significantly lower than in animals raised on a normal diet.

Zebrafish is arguably one of the most powerful models to study adult neurogenesis. This is due to robust proliferative capacity of zebrafish brain, with neural stem cells localized to 16 discrete niches along its entire rostro-caudal axis [78]. Similar to that in mammals, the neurons and glial cells produced are specific to neurogenic niches [79]. Our recent finding that adult neurogenesis in zebrafish is under robust control of the circadian system [56], with intriguing inter-niche differences, is the first direct evidence of the interaction between the clock and adult neurogenesis in diurnal vertebrates.

Here, we demonstrate that adult neurogenesis is dramatically reduced in 1-year old HCI fish. This effect is significantly more pronounced in HCI animals than in aged 3-year old fish receiving a regular amount of food. The low regenerative capacity of HCI brain may result from accelerated aging of the overall organism and altered metabolic processes that modulate the cell division cycle. However, more needs to be learned regarding the cause-effect relationship, since deficient neurogenesis may impair brain functions and thus interfere with the normal regulation of peripheral tissues and organs, circadian clock, sleep, and behavior. When considering an emerging role of diet in the development of neurodegenerative disorders in humans [80], this opens further opportunities to investigate the role of the interaction between the circadian factors, sleep, and metabolism in “successful” and “unsuccessful” aging of brain functions.

Together, a complex combination of alterations resulting from chronic high caloric intake, its similarity with changes observed in normally aging zebrafish, and the high throughput capability of zebrafish model can significantly assist in exploring the effects of diet and its specific components on the aging process. This vertebrate model can also significantly promote the search for drugs slowing down the aging process and improving the metabolic state of the organism after chronic exposure to maladaptive diets.

## 4. Materials and Methods

### 4.1. Animals

Adult male zebrafish (Danio rerio, wild-type AB strain (Catalog ID ZL1, Zebrafish International Resource Center (ZIRC), Eugene, OR, USA), 1–4 years of age, were raised in laboratory conditions, and maintained on a 14 h light:10 h dark (14:10 LD) cycle, at 28 °C, in 3 L tanks of a multi-tank system (Aquaneering, San Diego, CA, USA), as per standard practices [81]. Control populations studied under regular feeding conditions included 162 1-year old, 87 2-year old, 67 3-year old, and 56 4-year old fish. Populations of fish raised on chronic high caloric intake (HCI) included 205 1-year old and 132 2-year old fish. Housing the Control and HCI fish on the same multi-tank and multi-rack recirculating system, daily water changes, and tank sanitation assured similar optimal environment for all of the populations studied. All of the animal procedures were performed in accordance with Institutional Animal Care and Use Committee (IACUC) of Boston University School of Medicine (Protocol AN14366, 30 October 2015).

### 4.2. Feeding

Embryos hatched at 3–4 days post fertilization (dpf) and feeding started at 5 dpf. At 5–12 dpf, larvae were fed paramecium, and at 13–20 dpf—Type L saltwater rotifers (Brachionus plicatilis), both ad libitum. Thereafter, the Control animals were fed twice-a-day, at zeitgeber times (ZT) one and eight (ZT0 = lights on time), with Gemma-300 pellets (Skretting, Westbrook, ME, USA) and supplemental live feed of Artemia salina nauplii/metanauplii (brine shrimp). Total weight of daily food available to each animal was equal to approximately 1.7% of body weight, with brine shrimp constituting approximately 20% of total food received. The second age-matched group of fish was maintained on high caloric diet and, while on a similar to Control feeding schedule, they received Gemma-300 pellets at approximately 5% body weight per day. Visual observations of the time animals spent feeding indicated a longer period of food consumption in HCI and no difference in the amount of food leftovers on the floor of the tank within an hour after food administration, suggesting a higher food intake. For two months prior to the experimental procedures described in this paper, all of the fish involved in this study remained on the Control diet. Gemma feed contained 300 micron food pellets, with 59% protein, 14% lipid, 0.2% fiber, 1.3% phosphates, and 14% ash. Brine shrimp are, on average, 450 micron long and contain 54% protein, 21% lipid, 19% carbohydrate, and 12% ash.

### 4.3. Behavioral Activity Recordings

Sleep and locomotor activity patterns were documented on a flow-through high-throughput experimental system. The system allows the studying of up to 24 fish, in parallel, under controlled conditions of daily light-dark cycle (14:10 LD; 150 lux:0). The video tracking (ViewPoint, Montreal, QC, Canada) was conducted in infrared (IR) light, using IR-sensitive cameras equipped with IR pass filters to exclude variation due to ambient light cycle. Individual 1 L housing tanks (100 mm water column) were placed on an experimental rack, with the lateral wall of each tank facing the camera. The walls of adjacent individual tanks were white and non-transparent. The fish were habituated to experimental conditions prior to the initiation of the recording, and were fed as per regular schedule. Locomotor activity in the entire tank and within each of three equally-spaced areas of the tank (the top, middle, and bottom) was documented continuously, at 10 frames/s, with automatic 15-s integration period. For analysis, the duration and speed of locomotion (high, low, inactivity), total and at each area of the tank, was presented as mean (SEM) for consecutive 1-h intervals. Sleep was defined based on the number of 15-s epochs with activity level not exceeding 5% of mean daily activity level for a given animal. Based on our earlier observations, this corresponds to the 5–6 s intervals of inactivity associated with changes in arousal threshold indicative of sleep in zebrafish [42,69,71]. Feeding behavior and its duration was monitored visually, based on characteristic body position, body, and mouth movements, which is known to be indicative of zebrafish foraging [82]. Behavioral studies involved 8–12 fish per age or food intake group, documented in parallel.

### 4.4. Treatments

#### 4.4.1. 5-Ethynyl-2′-deoxyuridine (EdU) Treatment

For a 2 h interval at ZT9-11, individual groups (*n* = 6 per 1 L tank) were injected intraperitoneal (i.p.) injection with S-phase marker 5-ethynyl-2′-deoxyuridine (EdU; Molecular Probes, Eugene, OR, USA) in 0.1 M PBS at 50 μg/g, as per earlier report [83].

#### 4.4.2. Diazepam

Diazepam (DzP, Abbott Laboratories, Chicago, IL, USA) working solution was 17.5 mM and contained 10% ethanol in water. An earlier established [53] non-sedative dose of diazepam (5 μM final concentration in the fish tank) or control solution (0.003% ethanol) was administered at ZT4 and behavior monitored for the next 4 h in HCI and Control fish, in parallel.

### 4.5. EdU Staining

Fish were euthanized through submersion in ice water for 10 min until operculum movements ceased. Heads were fixed at 4 °C overnight in 4% paraformaldehyde/0.1 M phoshate buffered saline (PBS). Brains were dissected out, transferred for cryoprotection in 30% sucrose/0.1 M PBS, embedded in optimal cutting temperature (O.C.T.) compound and stored at −80 °C. Brains were cut on a cryostat (Microm HM505E, Walldorf, Germany) in 20 μm coronal sections and placed on slides stored at −80 °C until processing. The slides were washed in 0.1 M PBS and incubated in Click-iT^®^ reaction cocktail for 30 min from the Click-iT^®^ EdU Alexa Fluor^®^ 488 Flow Cytometry Assay (Invitrogen, Carlsbad, CA USA). After rinsing in PBS, slides were mounted using Vectashield mounting medium (Vector, Burlingame, CA, USA).

### 4.6. Microscopy, Analysis and Brain Volume Estimates

Images were taken using a Zeiss LSM 710 Observer Z1 inverted confocal microscope (Oberkocken, Germany) Using Zen software (Zeiss, Oberkochen, Germany), images were acquired with a 20× objective. Sequential image acquisition was performed. The total number of labeled cells was determined in the entire cerebellar neurogenic niche according to [78], using the Volocity software 6.3 (PerkinElmer Improvision, Waltham, MA, USA), after extensive validation through manual cell counting. To account for inter-individual difference in brain size, the data were adjusted for brain volume, which was estimated to be based on images of individual brain sections (ImageJ, National Institutes of Health, Bethesda, MD, USA) and using the Cavalieri principle [84]. This also served for the comparison of brain volumes between age matched Control and HCI fish at 1 years of age.

### 4.7. Real-Time Quantitative RT-PCR

Fish (*n* = 6) at each time point were collected via submersion in liquid nitrogen. Brains were removed and stored at −80 °C. Total RNA was isolated with the use of QIAzol Lysis reagent and RNAeasy kit (Qiagen, Hilden, Germany). RNA from each sample was converted into cDNA using the High-Capacity cDNA Archive kit (Applied Biosystems, Foster City, CA, USA). Quantitative RT-PCR (qPCR) was performed using a TaqMan Universal or SYBR Green PCR Master Mix and ABI Prism 7300 Real Time PCR System (Applied Biosystems). The probes and primers were based on the following sequences as described in [56]: *cyclin A2* (forward:5′-TGG AGA ACA ACC AGA GGA GAC A-3′, reverse: 5′-GCA TTT TCTTCAGGTTTACACGCAAT-3′, {CCAGACCCCTGTTTA GC}); *cyclin B2* (forward: 5′-GCG AAC TGT CTA ATC TTT CCC ACA A-3′, reverse: 5′-CGG CCA GTG GGT TTT ACA C-3′, {CAG TTC AGA CAA AGA AGG TT}); *Bmal1* (forward: 5′-CAG AGC TTC GCC ACA AAC C-3′, reverse: 5′-CTG TGA TCA ATG CAT GTC CTT TCA-3′, {CTC GAT GTG AGG ATC TG}); *Clock1* (forward: 5′-CAT CCT ACA GAA GAG CAT CGA CTT-3′, reverse: 5′-GAT TTC ACT CGA CTC CGA CTG T-3′, {AAG CAC AAA GAA ATT G}); *Per1* (forward: 5′-ATT CCG CCT AAC CCC GTA TGT GAC C-3′, reverse: 5′-GTG TGC CGC GTA GTG AAA ATC CTC TTG T-3′); *p20* (forward: 5′-GGT CCG TGT GGA CTT GAT TT-3′, reverse: 5′-CCT CTT CAA CAG CCC ATG AT-3′).

Gene expression was normalized for two housekeeping genes: *β-actin* and *EF1a* expression level for each individual fish sample: *β-actin* (forward: 5′-GCT GTT TTC CCC TCC ATT GTT G-3′, reverse: 5′-TTT CTG TCC CAT GCC AAC CA-3′; {CCC AGA CAT CAG GGA GTG}); *EF1a* (forward: 5′-GCA CGG TGA CAA CAT GCT-3′, reverse: 5′-TCC TTG CGC TCA ATC TTC CAT-3′; {ACC AGC CCA TGT TTG AG}).

### 4.8. Statistical Analysis

Using IBM SPSS Statistics software (SPSS, IBM Corp., Armonk, NY, USA), a one-way analysis of variance (ANOVA) with Tukey post *hoc* analysis was employed for comparison between the age and feeding groups for scoliosis, behavioral, and IHC measures. The same software was used for the analysis of gene expression, using two-way ANOVA (feeding × time), with Tukey post hoc analyses. Sleep data were analyzed using linear mixed model analysis (SPSS). The best-fit 24-h curves for the feeding and age groups were characterized using a least-squares sine wave fit to gene expression and sleep data (Mathematica, Wolfram, IL, USA). The resulting amplitude and phase estimates were compared using peak (acrophase) and 2-way ANOVA (time × group). Unless otherwise indicated, the significance level in all of the tests was set at *p* < 0.05.

## Abbreviations

HCI	High caloric intake
DPF	Days post fertilization
EdU	5-Ethynyl-2′-deoxyuridine
CDC	Cell division cycle
i.p.	Intraperitoneal
PBS	Phosphate-buffered saline
LD	Light-dark
ZT	Zeitgeber time
S-phase	Synthesis phase
SEM	Standard error of the mean

## References

[1] Poirier P., Giles T.D., Bray G.A., Hong Y., Stern J.S., Pi-Sunyer F.X., Eckel R.H. (2006). Obesity and cardiovascular disease: Pathophysiology, evaluation, and effect of weight loss: An update of the 1997 American Heart Association Scientific Statement on Obesity and Heart Disease from the Obesity Committee of the Council on Nutrition, Physical Activity, and Metabolism. Circulation.

[2] Guh D.P., Zhang W., Bansback N., Amarsi Z., Birmingham C.L., Anis A.H. (2009). The incidence of co-morbidities related to obesity and overweight: A systematic review and meta-analysis. BMC Public Health.

[3] Yaturu S. (2011). Obesity and type 2 diabetes. J. Diabetes Mellit..

[4] Jensen M.D., Ryan D.H., Apovian C.M., Ard J.D., Comuzzie A.G., Donato K.A., Hu F.B., Hubbard V.S., Jakicic J.M., Kushner R.F. (2014). 2013 AHA/ACC/TOS guideline for the management of overweight and obesity in adults: A report of the American College of Cardiology/American Heart Association Task Force on Practice Guidelines and The Obesity Society. Circulation.

[5] Akinnusi M.E., Saliba R., Porhomayon J., El-Solh A.A. (2012). Sleep disorders in morbid obesity. Eur. J. Intern. Med..

[6] Drager L.F., Togeiro S.M., Polotsky V.Y., Lorenzi-Filho G. (2013). Obstructive sleep apnea: A cardiometabolic risk in obesity and the metabolic syndrome. J. Am. Coll. Cardiol..

[7] Sohal R.S., Weindruch R. (1996). Oxidative stress, caloric restriction, and aging. Science.

[8] Anson R.M., Guo Z., de Cabo R., Iyun T., Rios M., Hagepanos A., Ingram D.K., Lane M.A., Mattson M.P. (2003). Intermittent fasting dissociates beneficial effects of dietary restriction on glucose metabolism and neuronal resistance to injury from calorie intake. Proc. Natl. Acad. Sci. USA.

[9] Bishop N.A., Guarente L. (2007). Two neurons mediate diet-restriction-induced longevity in *C. elegans*. Nature.

[10] Arslan-Ergul A., Ozdemir T.A., Adams M.M. (2013). Aging, Neurogenesis, and Caloric Restriction in Different Model Organisms. Aging Dis..

[11] Patel S.R., Hu F.B. (2008). Short sleep duration and weight gain: A systematic review. Obesity.

[12] Cappuccio F.P., Taggart F.M., Kandala N.-B., Currie A., Peile E., Stranges S., Miller M.A. (2008). Meta-analysis of short sleep duration and obesity in children and adults. Sleep.

[13] Chaput J.P., Després J.P., Bouchard C., Tremblay A. (2007). Association of sleep duration with type 2 diabetes and impaired glucose tolerance. Diabetologia.

[14] Nedeltcheva A.V., Scheer F.A.J.L. (2014). Metabolic effects of sleep disruption, links to obesity and diabetes. Curr. Opin. Endocrinol. Diabetes Obes..

[15] Spiegel K., Knutson K., Leproult R., Tasali E., Van Cauter E. (2005). Sleep loss: A novel risk factor for insulin resistance and Type 2 diabetes. J. Appl. Physiol..

[16] Knutson K.L. (2010). Sleep duration and cardiometabolic risk: A review of the epidemiologic evidence. Best Pract. Res. Clin. Endocrinol. Metab..

[17] Dashti H.S., Scheer F.A., Jacques P.F., Lamon-Fava S., Ordovás J.M. (2015). Short sleep duration and dietary intake: Epidemiologic evidence, mechanisms, and health implications. Adv. Nutr..

[18] Keckeis M., Lattova Z., Maurovich-Horvat E., Beitinger P.A., Birkmann S., Lauer C.J., Wetter T.C., Wilde-Frenz J., Pollmächer T. (2010). Impaired glucose tolerance in sleep disorders. PLoS ONE.

[19] Knutson K.L., Van Cauter E., Zee P., Liu K., Lauderdale D.S. (2011). Cross-sectional associations between measures of sleep and markers of glucose metabolism among subjects with and without diabetes: The Coronary Artery Risk Development in Young Adults (CARDIA) Sleep Study. Diabetes Care.

[20] Rudic R.D., McNamara P., Curtis A.M., Boston R.C., Panda S., Hogenesch J.B., Fitzgerald G.A. (2004). BMAL1 and CLOCK, two essential components of the circadian clock, are involved in glucose homeostasis. PLoS Biol..

[21] Turek F.W., Joshu C., Kohsaka A., Lin E., Ivanova G., McDearmon E., Laposky A., Olson S., Easton A., Jensen D.R. (2005). Obesity and metabolic syndrome in circadian Clock mutant mice. Science.

[22] Scott E.M., Carter A.M., Grant P.J. (2008). Association between polymorphisms in the Clock gene, obesity and the metabolic syndrome in man. Int. J. Obes..

[23] Sookoian S., Gemma C., Gianotti T.F., Burgueño A., Castaño G., Pirola C.J. (2008). Genetic variants of Clock transcription factor are associated with individual susceptibility to obesity. Am. J. Clin. Nutr..

[24] Uemura H., Katsuura-Kamano S., Yamaguchi M., Arisawa K., Hamajima N., Hishida A., Kawai S., Oze I., Shinchi K., Takashima N. (2015). A variant of the CLOCK gene and related haplotypes are associated with the prevalence of type 2 diabetes in the Japanese population. J. Diabetes.

[25] Corella D., Asensio E.M., Coltell O., Sorlí J.V., Estruch R., Martínez-González M.Á., Salas-Salvadó J., Castañer O., Arós F., Lapetra J. (2016). CLOCK gene variation is associated with incidence of type-2 diabetes and cardiovasculardiseases in type-2 diabetic subjects: Dietary modulation in the PREDIMED randomized trial. Cardiovasc. Diabetol..

[26] Garaulet M., Smith C.E., Gomez-Abellán P., Ordovás-Montañés M., Lee Y.C., Parnell L.D., Arnett D.K., Ordovás J.M. (2014). REV-ERB-ALPHA circadian gene variant associates with obesity in two independent populations: Mediterranean and North American. Mol. Nutr. Food Res..

[27] Patel S.A., Chaudhari A., Gupta R., Velingkaar N., Kondratov R.V. (2016). Circadian clocks govern calorie restriction-mediated life span extension through BMAL1- and IGF-1-dependent mechanisms. FASEB J..

[28] Katewa S.D., Akagi K., Bose N., Rakshit K., Camarella T., Zheng X., Hall D., Davis S., Nelson C.S., Brem R.B. (2016). Peripheral Circadian Clocks Mediate Dietary Restriction-Dependent Changes in Lifespan and Fat Metabolism in Drosophila. Cell Metab..

[29] Etienne C. (2007). Minireview: Entrainment of the Suprachiasmatic Clockwork in Diurnal and Nocturnal Mammals. Endocrinology.

[30] Kumar J.P., Challet E., Kalsbeek A. (2015). Circadian rhythms in glucose and lipid metabolism in nocturnal and diurnal mammals. Mol. Cell. Endocrinol..

[31] Beydoun M.A., Gary T.L., Caballero B.H., Lawrence R.S., Cheskin L.J., Wang Y. (2008). Ethnic differences in dairy and related nutrient consumption among US adults and their association with obesity, central obesity, and the metabolic syndrome. Am. J. Clin. Nutr..

[32] Whitmer R.A., Gustafson D.R., Barrett-Connor E., Haan M.N., Gunderson E.P., Yaffe K. (2008). Central obesity and increased risk of dementia more than three decades later. Neurology.

[33] Ashrafian H., Harling H., Darzi A., Athanasiou T. (2013). Neurodegenerative disease and obesity: What is the role of weight loss and bariatric interventions?. Metab. Brain Dis..

[34] Procaccini C., Santopaolo M., Faicchia D., Colamatteo A., Formisano L., de Candia P., Galgani M., De Rosa V., Matarese G. (2016). Role of metabolism in neurodegenerative disorders. Metabolism.

[35] Lee J., Duan W., Long J.M., Ingram D.K., Mattson M.P. (2000). Dietary restriction increases the number of newly generated neural cells, and induces BDNF expression, in the dentate gyrus of rats. J. Mol. Neurosci..

[36] Lee J., Seroogy K.B., Mattson M.P. (2000). Dietary restriction enhances neurotrophin expression and neurogenesis in the hippocampus of adult mice. J. Neurochem..

[37] Lee J., Duan W., Mattson M.P. (2000). Evidence that brain-derived neurotrophic factor is required for basal neurogenesis and mediates, in part, the enhancement of neurogenesis by dietary restriction in the hippocampus of adult mice. J. Neurochem..

[38] Bondolfi L., Ermini F., Long J.M., Ingram D.K., Jucker M. (2004). Impact of age and caloric restriction on neurogenesis in the dentate gyrus of C57BL/6 mice. Neurobiol. Aging.

[39] Kumar S., Parkash J., Kataria H., Kaur G. (2009). Interactive effect of excitotoxic injury and dietary restriction on neurogenesis and neurotrophic factors in adult male rat brain. Neurosci. Res..

[40] Lindqvist A., Mohapel P., Bouter B., Frielingsdorf H., Pizzo D., Brundin P., Erlanson-Albertsson C. (2006). High-fat diet impairs hippocampal neurogenesis in male rats. Eur. J. Neurol..

[41] Stangl D., Thuret S. (2009). Impact of diet on adult hippocampal neurogenesis. Genes Nutr..

[42] Hirayama J., Kaneko M., Cardone L., Cahill G., Sassone-Corsi P. (2005). Analysis of circadian rhythms in zebrafish. Methods Enzymol..

[43] Zhdanova I.V. (2006). Sleep in zebrafish. Zebrafish.

[44] Kishi S., Slack B.E., Uchiyama J., Zhdanova I.V. (2009). Zebrafish as a genetic model in biological and behavioral gerontology: Where development meets aging in vertebrates—A mini-review. Gerontology.

[45] Gerhard G.S., Kauffman E.J., Wang X., Stewart R., Moore J.L., Kasales C.J., Demidenko E., Cheng K.C. (2002). Life spans and senescent phenotypes in two strains of Zebrafish (*Danio rerio*). Exp. Gerontol..

[46] Tsai S.B., Tucci V., Uchiyama J., Fabian N.J., Lin M.C., Bayliss P.E., Neuberg D.S., Zhdanova I.V., Kishi S. (2007). Differential effects of genotoxic stress on both concurrent body growth and gradual senescence in the adult zebrafish. Aging Cell.

[47] Kishi S., Bayliss P.E., Uchiyama J., Koshimizu E., Qi J., Nanjappa P., Imamura S., Islam A., Neuberg D., Amsterdam A. (2008). The identification of zebrafish mutants showing alterations in senescence-associated biomarkers. PLoS Genet..

[48] Kacprzak V., Patel N.A., Riley E., Yu L., Yeh J.R., Zhdanova I.V. (2017). Dopaminergic control of anxiety in young and aged zebrafish. Pharmacol. Biochem. Behav..

[49] Yu L., Tucci V., Kishi S., Zhdanova I.V. (2006). Cognitive aging in zebrafish. PLoS ONE.

[50] Gilbert M.J., Zerulla T.C., Tierney K.B. (2014). Zebrafish (*Danio rerio*) as a model for the study of aging and exercise: Physical ability and trainability decrease with age. Exp. Gerontol..

[51] Zhdanova I.V., Yu L., Lopez-Patino M., Shang E., Kishi S., Guelin E. (2008). Aging of the circadian system in zebrafish and the effects of melatonin on sleep and cognitive performance. Brain Res. Bull..

[52] Levin E.D., Bencan Z., Cerutti D.T. (2007). Anxiolytic effects of nicotine in zebrafish. Physiol. Behav..

[53] López-Patiño M.A., Yu L., Cabral H., Zhdanova I.V. (2008). Anxiogenic effects of cocaine withdrawal in zebrafish. Physiol. Behav..

[54] Egan R.J., Bergner C.L., Hart P.C., Cachat J.M., Canavello P.R., Elegante M.F., Elkhayat S.I., Bartels B.K., Tien A.K., Tien D.H. (2009). Understanding behavioral and physiological phenotypes of stress and anxiety in zebrafish. Behav. Brain Res..

[55] Stewart A., Gaikwad S., Kyzar E., Green J., Roth A., Kalueff A.V. (2012). Modeling anxiety using adult zebrafish: A conceptual review. Neuropharmacology.

[56] Akle V., Stankiewicz A.J., Kharchenko V., Yu L., Kharchenko P.V., Zhdanova I.V. (2017). Circadian Kinetics of Cell Cycle Progression in Adult Neurogenic Niches of a Diurnal Vertebrate. J. Neurosci..

[57] Mitchell N., Catenacci V., Wyatt H.R., Hill J.O. (2011). Obesity: Overview of an epidemic. Psychiatr. Clin. N. Am..

[58] Thorpe R.J., Ferraro K.F. (2004). Aging, Obesity, and Mortality Misplaced Concern about Obese Older People?. Res. Aging.

[59] Chan J.S., Yan J.H., Payne V.G. (2013). The Impact of Obesity and Exercise on Cognitive Aging. Front. Aging Neurosci..

[60] Brown C.L., Halvorson E.E., Cohen G.M., Lazorick S., Skelton J.A. (2015). Addressing Childhood Obesity: Opportunities for Prevention. Pediatr. Clin. N. Am..

[61] Gurnani M., Birken C., Hamilton J. (2015). Childhood Obesity: Causes, Consequences, and Management. Pediatr. Clin. N. Am..

[62] Barzilai N., Huffman D.M., Muzumdar R.H., Bartke A. (2012). The critical role of metabolic pathways in aging. Diabetes.

[63] Maury E., Hong H.K., Bass J. (2014). Circadian disruption in the pathogenesis of metabolic syndrome. Diabetes Metab..

[64] Sato S., Solanas G., Peixoto F.O., Bee L., Symeonidi A., Schmidt M., Brenner C., Masri S., Benitah S.A., Sassone-Corsi P. (2017). Circadian Reprogramming in the Liver Identifies Metabolic Pathways of Aging. Cell.

[65] Hood S., Amir S. (2017). Neurodegeneration and the Circadian Clock. Front. Aging Neurosci..

[66] Bass J., Sassone-Corsi P., Christen Y. (2016). Circadian Mechanisms in Bioenergetics and Cell Metabolism. A Time for Metabolism and Hormones.

[67] Tamai T.K., Carr A.J., Whitmore D. (2005). Zebrafish circadian clocks: Cells that see light. Biochem. Soc. Trans..

[68] Longo V.D., Panda S. (2016). Fasting, circadian rhythms, and time-restricted feeding in healthy lifespan. Cell Metab..

[69] Mander B.A., Winer J.R., Walker M.P. (2017). Sleep and Human Aging. Neuron.

[70] Zhdanova I.V., Wang S.Y., Leclair O.U., Danilova N.P. (2001). Melatonin promotes sleep-like state in zebrafish. Brain Res..

[71] Levitas-Djerbi T., Appelbaum L. (2017). Modeling sleep and neuropsychiatric disorders in zebrafish. Curr. Opin. Neurobiol..

[72] Yokogawa T., Marin W., Faraco J., Pézeron G., Appelbaum L., Zhang J., Rosa F., Mourrain P., Mignot E. (2007). Characterization of sleep in zebrafish and insomnia in hypocretin receptor mutants. PLoS Biol..

[73] Appelbaum L., Wang G.X., Maro G.S., Mori R., Tovin A., Marin W., Yokogawa T., Kawakami K., Smith S.J., Gothilf Y. (2009). Sleep-wake regulation and hypocretin-melatonin interaction in zebrafish. Proc. Natl. Acad. Sci. USA.

[74] Elbaz I., Zada D., Tovin A., Braun T., Lerer-Goldshtein T., Wang G., Mourrain P., Appelbaum L. (2016). Sleep-Dependent Structural Synaptic Plasticity of Inhibitory Synapses in the Dendrites of Hypocretin/Orexin Neurons. Mol. Neurobiol..

[75] Chen A., Singh C., Oikonomou G., Prober D.A. (2017). Genetic Analysis of Histamine Signaling in Larval Zebrafish Sleep. eNeuro.

[76] Oikonomou G., Prober D.A. (2017). Attacking sleep from a new angle: Contributions from zebrafish. Curr. Opin. Neurobiol..

[77] Elbaz I., Levitas-Djerbi T., Appelbaum L. (2017). The Hypocretin/Orexin Neuronal Networks in Zebrafish. Curr. Top. Behav. Neurosci..

[78] Grandel H., Kaslin J., Ganz J., Wenzel I., Brand M. (2006). Neural stem cells and neurogenesis in the adult zebrafish brain: Origin, proliferation dynamics, migration and cell fate. Dev. Biol..

[79] Zupanc G.K., Hinsch K., Gage F.H. (2005). Proliferation, migration, neuronal differentiation, and long-term survival of new cells in the adult zebrafish brain. J. Comp. Neurol..

[80] Vauzour D., Camprubi-Robles M., Miquel-Kergoat S., Andres-Lacueva C., Bánáti D., Barberger-Gateau P., Bowman G.L., Caberlotto L., Clarke R., Hogervorst E. (2017). Nutrition for the ageing brain: Towards evidence for an optimal diet. Ageing Res. Rev..

[81] Whitmore D., Foulkes N.S., Sassone-Corsi P. (2000). Light acts directly on organs and cells in culture to set the vertebrate circadian clock. Nature.

[82] Spence R., Gerlach G., Lawrence C., Smith C. (2008). The behaviour and ecology of the zebrafish, *Danio rerio*. Biol. Rev. Camb. Philos. Soc..

[83] Zeng C., Pan F., Jones L.A., Lim M.M., Griffin E.A., Sheline Y.I., Mintun M.A., Holtzman D.M., Mach R.H. (2010). Evaluation of 5-ethynyl-2′-deoxyuridine staining as a sensitive and reliable method for studying cell proliferation in the adult nervous system. Brain Res..

[84] Prakash Y.S., Smthison K.G., Sieck G.C. (1994). Application of the Cavalieri principle in volume estimation using laser confocal microscopy. Neuroimage.

